# Tocilizumab administration in COVID-19 patients: Water on the fire or gasoline?

**DOI:** 10.1016/j.mmcr.2021.01.002

**Published:** 2021-01-21

**Authors:** Cristian Deana, Luigi Vetrugno, Flavio Bassi, Amato De Monte

**Affiliations:** a Department of Anesthesia and Critical Care, Academic Hospital of Udine, Udine, Italy; b Department of Medical Area, University of Udine, Udine, Italy

**Keywords:** Tocilizumab, COVID-19, Pulmonary aspergillosis, Critical care, Immunosuppression

## Abstract

Tocilizumab is widely being used to treat COVID-19. Although reducing systemic inflammation, it also increases the risk for secondary infections as a result of the immunosuppression produced. We report the case of a 69-year-old patient admitted to the ICU with severe respiratory distress caused by COVID-19 pneumonia who developed pulmonary aspergillosis. On the basis of these findings, we suggest early testing for pulmonary aspergillosis in COVID-19 patients treated with tocilizumab.

## 1 Introduction

*Aspergillus* is an opportunistic fungus mainly affecting immunocompromised patients. A high rate of invasive pulmonary aspergillosis has been demonstrated among critically ill patients admitted to the ICU with severe influenza, which may contribute to their high odds of mortality [1]. In the early days of the Sars-CoV-2 pandemic, several case reports emerged from Wuhan of COVID-19-associated pulmonary aspergillosis (CAPA) [2]. Many risk factors for CAPA were recognized among critically ill COVID-19 patients, including lymphopenia, high levels of systemic pro-inflammatory cytokines, and the use of steroids [3].

Tocilizumab is a recombinant humanized monoclonal antibody against the interleukin-6 receptor. Initially, it showed promising results in reducing the severity of COVID-19 infection in critically ill patients. It dramatically reduces the potent inflammatory effects of IL-6 responsible for causing the lung damage and eventual multiorgan failure associated with COVID-19 [4]. A large retrospective study found that tocilizumab reduced the risk of death and the odds of needing mechanical ventilation in severe COVID-19 pneumonia [5]. However, subsequent findings from large randomized controlled trials raised doubts regarding these findings.

Here, we report the case of a critically ill patient who developed CAPA following tocilizumab administration.

## 2 Case

A 69-year-old man was admitted to the emergency department (ED) with fever and a cough which he had had for two days. His medical history included HBV-related liver cirrhosis, arterial hypertension and mild obesity (Body Mass Index 32). The man was calm and cooperative during his ED visit. His vital signs were: ABP 160/100 mmHg, SpO2 97% while breathing room air (PaO2/FIO2=303 mmHg), HR 75/min, and body temperature 36.7 °C. His white blood cell count was 3849/mm^3^, platelets were 62,000/mm^3^ (abnormal), and lymphocytes were 2140/mm^3^. C-reactive protein was 1.48 mg/L, procalcitonin (PCT) was 0.04 ng/ml, pro-adrenomedullin (pro-ADM) was 0.69 nMol/L, and IL-6 was 27.0 pg/mL (abnormal). A real-time PCR nasal swab tested positive for SARS-COV2.

The patient was then admitted to the infectious diseases ward (day 0) where he was treated with dexamethasone intravenously 6 mg per day. Given his worsening clinical situation, which necessitated increasing levels of oxygen provided by non-invasive ventilation using a full face mask, he was finally admitted to the ICU (day 8). In the ICU, the patient required immediate endotracheal intubation and invasive mechanical ventilation due to worsening dyspnoea associated with a PaO2/FIO2 of 135 mmHg. On his second day in the ICU (day 10), the patient's IL-6 levels reached 223.0 pg/mL (abnormal), thus the decision to administer tocilizumab that same day was taken (the patient was also being treated with entecavir for HBV-related cirrhosis).

Following tocilizumab administration, pre-emptive ceftobiprole treatment was started, 500 mg intravenously every 8 hours according to our internal protocol (day 10 until day 19). On day 15, the patient underwent an early percutaneous tracheostomy, as per our routine practice in COVID-19 patients, in order to facilitate easier weaning from mechanical ventilation.

On day 23, acyclovir (10 mg/kg BW) was administered intravenously every 8 hours to treat *Herpes* reactivation (HSV1-DNA detected in blood: 5197 copies/mL, abnormal). The same day, liposomal amphotericin B (3 mg/kg BW that was stopped after 30 days of therapy) was also given despite the absence of microbiological findings due to the suspicion of CAPA as suggested by the patient's high serum levels of β–D glucan (86.9 pg/ml, abnormal).

*Aspergillus fumigatus* was finally isolated from the bronchial aspirate on day 37, 22 days after tocilizumab administration and 12 days after the first positive result for serum β–D glucan.

Galactomannan levels in serum and alveolar fluid were tested on day 17, 20, 26 and 32. In the same days bronchoalveolar lavage fluid was also investigated through standard bacterial and fungal cultures and multiplex PCR filmarray. All these tests resulted negative for *Aspergillus.*

**Fig. 1:**
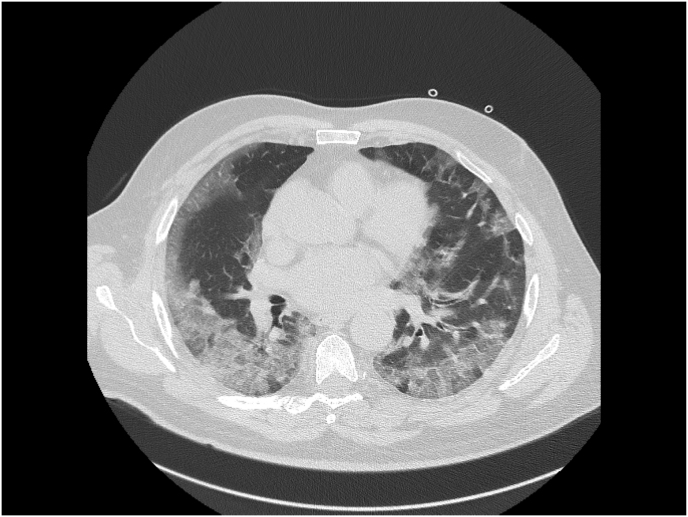
CT scan of the thorax at hospital admission (day 0) demonstrates diffuse subpleural ground-glass opacity.

**Fig. 2:**
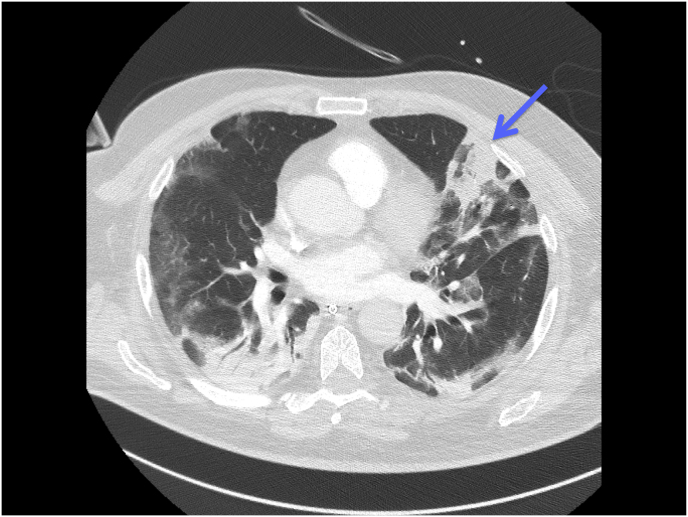
CT scan of the thorax taken on day 14 demonstrates the appearance of consolidation in the upper left pulmonary lobe (blue arrow), extended ground-glass opacity and the crazy-paving pattern associated with posterior lung consolidation. (For interpretation of the references to colour in this figure legend, the reader is referred to the Web version of this article.)

**Fig. 3:**
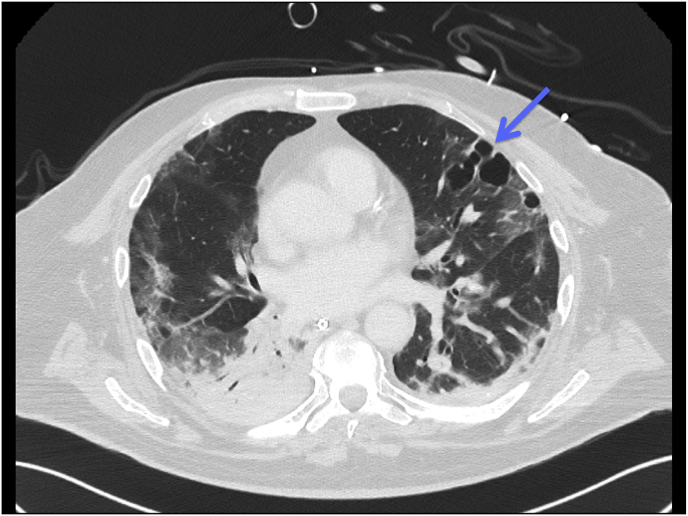
CT scan (day 28) demonstrating a large septated cavity in the lung parenchyma suspected to be an *Aspergillus* lesion (blue arrow). (For interpretation of the references to colour in this figure legend, the reader is referred to the Web version of this article.)

Repeated CT scans of the thorax showed the development over time of a cavity in the lung parenchyma, supporting our suspicion of pulmonary *Aspergillus* infection (see Fig. 1, Fig. 2, Fig. 3).

On day 43, the patient was discharged from the ICU to the Pneumology Unit with spontaneous breathing through the tracheostomy, which was removed on day 58. He went to rehabilitation unit in good condition after 68 days of hospitalization.

## 3 Discussion

The COVID-19 pandemic raises important questions regarding the treatment of coronaviruses. To the best of our knowledge, no single drug has been demonstrated to be effective in critically ill patients, and more evidence is required [6,7]. Many drugs and treatments (e.g. hydroxychloroquine, lopinavir/ritonavir, darunavir/cobicistat, etc.) have been proposed for the treatment of critically ill COVID-19 patients. One goal of these treatments is to reduce viral spread and replication. Another is to limit hyperinflammation and the triggering of a systemic response during the later phase of an infection which could lead to multiorgan dysfunction [8].

Steroids constitute the cornerstone treatment for COVID-19. The aim of steroid treatment is to modulate the inflammatory response, being recognized as a standard of care in the latest WHO guidelines [9]. Other drugs have also been proposed and tested for their capacity to limit the hyperinflammation that characterizes the later stages of COVID-19. These include inhibitors of the IL-1β and IL-6 receptor, of which anakinra and tocilizumab are the most commonly used. It is worth noting here that IL-6 also promotes viral and bacterial clearance. However, excessive inflammation could increase vascular permeability, inducing acute respiratory distress syndrome (ARDS), cardiac arrhythmia and other negative consequences [10].

From a theoretical perspective, tocilizumab therapy is currently considered the most appropriate. Its overall goal is to prevent multiorgan dysfunction caused by a cytokine storm. However, the associated consequences of immunosuppression must also be taken into account. Warnings about the increased risk of bacterial and fungal infections following immunosuppressant therapy have become more numerous over the last year [11]. Lymphopenia is present in a high percentage of COVID-19 patients. The additional use of steroids and other immunosuppressive drugs thus increases the risk for fungal infections, especially *Aspergillus* sp. In the case described, HBV-related cirrhosis was an additional risk factor for fungal infection. Indeed, evidence suggests there to be more secondary infections in COVID-19 patients treated with tocilizumab than in those not receiving the drug [12]. Moreover, it is difficult to confirm suspected infections using standard laboratory tests for infection, such as C-reactive protein or procalcitonin. These markers rapidly decrease after tocilizumab administration, with very low values persisting for many days. As a consequence, doctors are unlikely to suspect an infection.

Some authors recommend empirical antibiotic prophylaxis, including antifungal drugs. Indeed, the difficulty and time involved in isolating *Aspergillus* from bronchoalveolar lavage fluid further support the adoption of an empirical antifungal approach to COVID-19 therapy in critically ill patients [13]. In fact, in the case herein described, following our initial suspicion of CAPA due to the significant increase in β–D glucan levels (on day 23), the first microbiological specimen testing positive for *Aspergillus* was not obtained until 12 days later.

Diagnosis of invasive pulmonary aspergillosis (IPA) in the ICU remains challenging. For this reason, the European Organization for Research and Treatment of Cancer/Mycoses Study Group (EORTC/MSG) criteria have been supplemented with a specific algorithm in critically ill patients [14]. Although biomarkers of *Aspergillus* infection, such as serum galactomannan or β–D glucan, are generally deemed to be unsensitive, testing for their presence in serum and bronchoalveolar lavage fluid was recently added to the EORTC/MSG criteria to aid the identification of IPA in influenza patients admitted to ICU. In our case, in addition to the patient's risk factors for IPA, the sudden increase in β–D glucan levels was enough for us to deem therapy with a broad spectrum antifungal necessary. Curiously, although the patient tested positive for β–D glucan, he remained negative for galactomannan.

In the present case, tocilizumab may have favoured CAPA, although this remains to be proven.

Many questions remain unanswered regarding the role of tocilizumab therapy in COVID-19. Physicians generally prescribe it believing that they are damping down the fire … however, in some cases, it might be better likened to adding gasoline and not water!

## Declaration of competing interest

The authors have no conflicts of interest to declare.
